# Effects of the Convention on the Rights of the Child on child mortality and vaccination rates: a synthetic control analysis

**DOI:** 10.1186/s12914-019-0211-9

**Published:** 2019-08-02

**Authors:** Gary W. Reinbold

**Affiliations:** Department of Public Administration and Institute for Legal, Legislative, and Policy Studies, University of Illinois at Springfield, Springfield, IL 62703 USA

**Keywords:** Child mortality, Vaccination, Convention on the rights of the child, Human rights, Synthetic control methods

## Abstract

**Background:**

Scholars have long been sceptical about the effectiveness of human rights treaties in changing the behaviour of states parties and prior empirical research has often justified that scepticism. However, only a few prior studies have considered the effects of adoption of core human rights treaties on health outcomes and only one prior study has analysed the effects of adoption of the Convention on the Rights of the Child (CRC) on children’s health outcomes.

**Methods:**

In this study, we estimated the effects of CRC adoption on child mortality rates and vaccination rates in less developed countries. In particular, we compared 43 less developed countries that adopted the CRC in 1990 with synthetic control groups drawn from 21 less developed countries that adopted it after 1992.

**Results:**

We find that CRC adoption may be related to additional reductions in infant and under-5 mortality rates of about 1 to 2 deaths per 1000 live births, on average, during the first three years after adoption, although those relationships are not statistically significant. And we find that CRC adoption is related to additional increases in vaccination rates for the five vaccines that we considered of about 4 to 5%, on average, during the first three years after adoption and that those relationships remain significant for up to seven years after adoption.

**Conclusion:**

From a policy perspective, our results further support the effectiveness of CRC adoption in promoting children’s right to health in less developed countries. And from a research perspective, our results show the advantages of using synthetic control methods in these types of studies, because our analyses using other methods that have most commonly been used in these studies did not find any consistent, significant relationships between CRC adoption and mortality or vaccination rates.

## Background

The Convention on the Rights of the Child (CRC) is one of nine core international human rights treaties currently in effect and the only one that focuses entirely on children. The United Nations General Assembly adopted the CRC in 1989 and the CRC took effect on September 2, 1990, after 20 countries had ratified it. The CRC is now the most widely adopted human rights treaty, as 192 of the 193 United Nations member states are parties to it, along with both non-member observer states (the Holy See and the State of Palestine), the Cook Islands, and Niue; the United States is the only United Nations member state that is not a party to the CRC [1].

The CRC addresses a wide range of children’s rights, including civil and political rights and economic, social, and cultural rights. The economic and social rights include rights relating to health, social security, an adequate standard of living, and education. With respect to health, in particular, Article 24 of the CRC obligates states parties to take appropriate measures to diminish infant and child mortality, to provide necessary health care, to combat disease and malnutrition, and to develop preventive health care.

Ironically, the near universal adoption of the CRC has made it challenging to assess its effectiveness. Certainly, most indicators of child well-being have improved since the CRC became effective in 1990. Infant mortality and under-5 mortality have decreased by 53% and 56%, respectively [2]. The prevalence of under-5 stunting (more than two standard deviations below median height-for-age) has decreased by 56% [3]. Vaccination rates for the third dose of diphtheria, tetanus, and pertussis vaccine (DTP3), for the first dose of measles-containing-vaccine (MCV1), for the third dose of polio vaccine (Pol3), and for Baccille Calmette Guerin vaccine (BCG, to prevent tuberculosis) have increased by 11%, 12%, 10%, and 7%, respectively [4]. And access to basic water and sanitation have increased by 8% and 9%, respectively, since 2000 [5]. Of course, one cannot necessarily attribute this progress to the CRC. Most measures of child well-being have generally improved over time, even prior to the CRC. And other global efforts to improve the lives of children in the past 30 years may have contributed to this progress, notably the United Nations Children’s Fund’s universal childhood immunization campaign in 1985, the World Summit for Children in 1990, and the Millennium Development Goals in 2000.

Many country-specific studies have described particular legislative and administrative actions to implement the CRC that seem likely to have improved children’s rights [6–15]. Several of those studies have also noted corresponding improvements in some measures of child well-being in those countries, including vaccination rates [11]; female genital mutilation rates, adoptions, and juvenile detention and imprisonment rates [14]; child poverty and hunger rates, access to basic sanitation, school enrolment rates, and standardized test performance [9]; and child mortality and malnutrition rates [10]. But one cannot generalize the results of these studies to CRC states parties generally, nor can one even be certain that those changes would not have occurred in those specific countries absent their adoption of the CRC.

Over the past 20 years, many studies have estimated the effects on various outcomes of adoption of other core human rights treaties, including the International Covenant on Civil and Political Rights (ICCPR) [16–26], the Convention against Torture and Other Cruel, Inhuman or Degrading Treatment or Punishment (CAT) [16, 18–20, 22, 25–29], the Convention on the Elimination of All Forms of Discrimination against Women (CEDAW) [16, 20, 22, 26, 30, 31], the International Convention on the Elimination of All Forms of Racial Discrimination (CERD) [16, 26], and groups of those treaties considered collectively [32–34]. Those studies generally have not found significant effects of adoption of those treaties on the outcomes considered and, when they have, they have found significant harmful effects almost as often as significant beneficial effects. However, the results have varied by treaty, with analyses of the ICCPR generally finding no effects on civil and political rights, analyses of CEDAW generally finding beneficial effects on women’s rights, and analyses of CAT generally finding harmful effects on freedom from torture.

Only a few prior studies have considered the effects of adoption of core human rights treaties on health outcomes. Gray, Kittilson, and Sandholtz found significant beneficial effects of CEDAW ratification on female life expectancy [31]. Simmons found significant beneficial effects of CEDAW ratification on family planning access [26]. Palmer et al. did not find any significant associations between ratification of six core human rights treaties (CRC, the International Convention on Economic, Social and Cultural Rights (ICESCR), CEDAW, CAT, CERD, and ICCPR) and HIV prevalence, maternal mortality, infant mortality, under-5 mortality, or life expectancy [34]. And, in the only prior study to consider the individual effects of CRC adoption on any outcomes, Simmons also found that CRC ratification had weakly significant, beneficial effects on MCV1 vaccinations and no significant effects on DTP3 vaccinations [26]. The fact that the CRC has been almost universally adopted has likely dissuaded researchers from considering it as often as other core human rights treaties (“Most countries have. ratified the convention on the rights of the child, thus making any comparison useless.” ([34], p., 1991)). It is not clear why so few studies have analysed the effects on health outcomes of other core human rights treaties, such as CEDAW and the ICESCR.

A principal challenge in any analysis of the effects of adoption of human rights treaties is addressing the potential selection effects. Countries that adopt a human rights treaty may already be more committed to improving relevant outcomes under the treaty, making it difficult to determine whether any improvement in those outcomes is due to the country’s adoption of the treaty or to the country’s pre-existing commitment that led it to adopt the treaty. Some analyses have attempted to address these potential selection effects with either instrumental variables [16, 17, 26, 27] or propensity score or similar matching methods [20, 22–24]. Most analyses have also included lagged dependent variables, which control for the country’s pre-existing commitment to some extent [17, 19, 21–26, 30, 32, 33]. Of these approaches, matching methods seem most promising, because of the difficulty of identifying valid instruments. However, applying matching methods to the CRC is difficult, because most countries adopted it within a few years after its effective date, thus leaving few untreated units for matching.

In this study, we attempted to estimate the effects of CRC adoption on child mortality and vaccination rates by focusing on those first few years after its effective date, when there was maximal variability in its adoption status across countries. We did so using synthetic control methods, a more advanced matching technique developed by Abadie and Gardeazabal [35] and Abadie, Diamond, and Hainmueller [36] and extended to panels with multiple treated units by Cavallo, Galiani, Noy, and Pantano [37]. Specifically, we sought to determine whether countries that adopted the CRC immediately in 1990 achieved larger reductions in child mortality rates and increases in vaccination rates over the next several years than similar countries that could have adopted the CRC in 1990, but did not do so until at least 1993.

## Methods

We needed to identify a time period with maximal variability in terms of countries’ adoption of the CRC. Throughout this article, we use the word “adopted” to indicate that the country became legally bound under the treaty, whether through ratification, acceptance, accession, or succession; adoption does not include merely signing the treaty without ratification. The first column of Table 1 shows that about one-third of countries (62) adopted the CRC in 1990, the year in which it became effective; another one-third of countries (62) adopted the CRC in 1991 or 1992; and another one-third of countries (70) adopted the CRC in 1993 or later. (In addition to the 192 United Nations member states that have adopted the CRC, Table 1 includes the Cook Islands and Niue; it does not include the Holy See or the State of Palestine.) Therefore, we compared the group that adopted in 1990 (the treatment group) with the group that adopted in 1993 or later (the control group); we would have preferred a longer time difference between the two groups’ adoption of the CRC, but that would have left too few countries in the control group. Because we wanted to compare similar countries, we further limited the control group to countries that existed in 1990; the second column of Table 1 shows the numbers of those countries by adoption year. And, because we expect any effects of CRC adoption on child mortality and vaccination rates to be larger in less developed countries, we further limited both groups to countries with under-5 mortality rates greater than 10 per 1000 live births in 1990; the third column of Table 1 shows the numbers of those countries by adoption year. Thus, 56 and 43 countries remained in the treatment and control groups, respectively.

**Table 1:** Adoption timing of the Convention on the Rights of the Child

Year	Number of countries adopting CRC	Number of those countries that existed in 1990	Number of those countries with under-5 mortality rates greater than 10 in 1990
1990	62	62	56
1991	42	39	33
1992	20	17	16
1993	28	22	20
1994	14	9	7
1995	17	16	11
1996	3	3	2
1997	3	3	2
2001	1	0	0
2003	1	0	0
2006	1	0	0
2015	2	1	1
Total	194	172	148

### Year fixed effect regressions

We first conducted year fixed effect regressions, both because prior studies have often used such regressions and because they were useful in selecting predictor variables for our synthetic control analyses. In these analyses, we included all countries with under-5 mortality rates greater than 10 per 1000 live births in 1990, regardless of their CRC adoption years. We used dependent variable values forwarded five years from the independent variable values, because we expect any effects of CRC adoption to be largest a few years after that adoption. In addition to the control variables described below, we included the year fixed effects to control for aggregate time trends (CRC adoption and vaccination rates increase over time, while mortality rates decrease over time). We also controlled for two values of the dependent variable, one from the same year as the independent variable value and one lagged five years from that value, to control for both the baseline level and the prior trend in the dependent variable. We limited these regressions to the period of maximal variability in CRC adoption status across countries, the period from 1990 to 1995.

Table 2 shows descriptive statistics for the variables included in our year fixed effect regressions. We considered seven dependent variables. *Infant mortality rate* measures the number of deaths by children younger than 1 year old per 1000 live births. *Under-5 mortality rate* measures the number of deaths by children younger than 5 years old per 1000 live births. *BCG vaccine rate* measures the percentage of live births who received the BCG vaccine. *DTP1 vaccine rate* measures the percentage of surviving infants who received the first dose of diphtheria, tetanus, and pertussis vaccine. *DTP3 vaccine rate* measures the percentage of surviving infants who received the DTP3 vaccine. *MCV1 vaccine rate* measures the percentage of surviving infants who received the MCV1 vaccine. *Polio3 vaccine rate* measures the percentage of surviving infants who received the Pol3 vaccine. All of these variables use data from the United Nations Children’s Fund [2, 4]. We would have preferred to include additional dependent variables measuring other dimensions of child well-being, but we could not find consistent, annual data on other indicators for these countries that extend back to at least 1985, as we needed for our analyses. For example, the World Bank data on child labour that Simmons used in her analysis is available only sporadically for these countries, especially prior to the 1990s; Simmons had to interpolate much of that data to support her analysis.

**Table 2:** Descriptive statistics for study variables from 1990 to 1995

Variable	Country-years	Mean	Standard deviation	Minimum	Maximum
Dependent variables:					
Infant mortality rate	984	54.7	38.5	6.2	171.2
Under-5 mortality rate	984	80.4	67.6	7.4	328.2
BCG vaccine rate	828	84.5	17.4	12	99
DTP1 vaccine rate	901	88.0	13.8	31	99
DTP3 vaccine rate	901	75.9	20.9	10	99
MCV1 vaccine rate	895	75.0	19.5	12	99
Polio3 vaccine rate	901	76.5	21.0	8	99
Independent variable:					
CRC adoption	940	0.72	0.45	0	1
Control variables:					
Ln (population)	935	15.3	2.1	9.1	20.9
Ln (GDP per capita)	851	7.0	1.2	4.2	10.2
Trade as percent of GDP	803	73.7	40.0	0.1	355.0
ODA as percent of GNI	722	10.6	16.0	0	242.3
Polity IV democracy index	814	3.7	3.8	0	10
Polity IV durability	814	13.5	16.9	0	88
Civil war	944	0.21	0.41	0	1
International war	944	0.03	0.18	0	1
Instruments:					
Simmons ratification hurdles index	563	1.75	0.63	0	3
DPI federalism index	828	0.32	0.33	0	1
Prior ICESCR adoption	944	0.60	0.49	0	1
Prior CEDAW adoption	944	0.65	0.48	0	1
Prior regional CRC adoption percent	944	0.55	0.32	0	1

Our independent variable was a dummy variable coded 1 if the country had adopted the CRC during or prior to the year in question and 0 otherwise. As control variables, we included several variables that have been used in other studies of the effects of human rights treaties. *Ln (population)*, *ln (GDP* per capita*)*, *trade as percent of GDP*, and *ODA as percent of GNI* are all from World Bank data [38–41]. *Polity IV democracy index* is the 10-point democracy index and *Polity IV durability* is the length of the country’s current regime, both from the Polity IV dataset [42]. *Civil war* is a dummy variable coded 1 if the corresponding variable was coded 1 in either the Correlates of War Project database [43] or the Uppsala Conflict Data Program/Peace Research Institute Oslo database [44] and 0 otherwise. Similarly, *international war* is a dummy variable coded 1 if the corresponding variable was coded 1 in either of those two databases and 0 otherwise.

Table 2 also includes descriptive statistics for several additional variables that we used as instruments in our instrumental variable analyses described below. *Simmons ratification hurdles index* is a four-point index developed by Simmons to measure the difficulty of a country’s treaty ratification process [45]. *DPI federalism index* is a five-category federalism index from the World Bank Database of Political Institutions [46]. *Prior ICESCR adoption* is a dummy variable indicating whether the country had previously adopted the ICESCR. *Prior CEDAW adoption* is a dummy variable indicating whether the country had previously adopted CEDAW. *Prior regional CRC adoption percent* measures the percentage of countries in the same world region that had previously adopted the CRC.

### Synthetic control analyses

Our main analyses used synthetic control methods to estimate the effects of CRC adoption on the dependent variables. These methods match each treated unit with a weighted combination of control units, selecting those control units and weights to match the treated unit’s values for the predictor variables as closely as possible [47]. We used the *synth_runner* program in Stata to implement the synthetic control methods for panel data with multiple treated units developed by Cavallo et al. [37]. As described above, the treatment group included countries that ratified the CRC in 1990 and the control group included countries that existed in 1990, but did not ratify the CRC until after 1992. Both groups were limited to countries with under-5 mortality rates greater than 10 per 1000 live births in 1990. As predictors, we included four variables that had significant relationships with the dependent variables in our year fixed effect regressions, as described in the Results section: *ln (population)*, *ln (GDP* per capita*)*, *ODA as percent of GNI*, and *Polity IV democracy index*; we also included the relevant dependent variable as a predictor. We matched the average values of those predictors between each treated unit and its synthetic control group over the period from 1985 to 1989. Table 3 presents descriptive statistics for the predictor variables used in our synthetic control analyses. As Table 3 shows, the treatment group countries were larger, poorer, and more democratic and had worse child outcomes, on average, than the control group countries.

**Table 3:** Descriptive statistics by group for synthetic control method predictor variables from 1985 to 1989

Predictor variable	Treatment group			Control group		
	Country-years	Mean	Standard deviation	Country-years	Mean	Standard deviation
Ln (population)***	215	16.2	1.4	105	15.2	1.7
Ln (GDP per capita)***	210	6.4	0.9	105	7.4	1.0
ODA as percent of GNI	206	7.7	7.8	105	6.3	9.6
Polity IV democracy index**	215	2.9	3.7	105	1.7	2.8
Infant mortality rate***	215	79.1	38.8	105	61.5	38.8
Under-5 mortality rate***	215	127.5	77.8	105	85.9	61.3
BCG vaccine rate***	209	67.0	24.0	95	84.2	15.1
DTP1 vaccine rate***	209	71.1	21.7	100	84.1	12.9
DTP3 vaccine rate***	209	51.3	25.6	100	69.0	18.4
MCV1 vaccine rate***	209	53.9	22.5	99	65.1	17.6
Polio3 vaccine rate***	209	53.9	27.4	100	69.1	18.8

*Note*. Differences between group means are significant as follows: ** *p* < .01; *** *p* < .001

## Results

### Year fixed effect regressions

Table 4 presents the results of our year fixed effect regressions. The relationships between CRC adoption and the five-year-forwarded values of the dependent variables were never quite significant, but were all in the expected direction. The effect sizes were rather small, with additional reductions of 1.2 and 2.5 in infant and under-5 mortality rates per 1000 live births, respectively, and additional increases in vaccination rates ranging from 1.0% to 2.3%. Among the control variables, four were significant at the 5% level in at least some of our year fixed effect regressions: *ln (population)*, *ln (GDP* per capita*)*, *ODA as percent of GNI*, and *Polity IV democracy index*. The same-period (year *t*) value of the dependent variable was always highly significant. The lagged (year *t - 5*) value of the dependent variable was also highly significant for infant mortality rates, but was rarely significant for other outcomes.

**Table 4:** Year fixed effect regression estimates of relationship between CRC adoption and child mortality and vaccination rates

Variables included in regression in year t (except as specified)	Dependent variable in year t + 5						
	Infant mortality rate	Under-5 mortality rate	BCG vaccine rate	DTP1 vaccine rate	DTP3 vaccine rate	MCV1 vaccine rate	Polio3 vaccine rate
CRC adoption	−1.15	−2.48	2.31	1.04	2.09	2.14	2.07
Ln (population)	−0.85*	−1.95*	0.96	0.96*	1.36*	1.50*	1.44*
Ln (GDP per capita)	−0.25	− 1.49	4.07***	2.00*	2.46+	3.84**	2.91*
Trade as percent of GDP	−0.00	− 0.00	−0.00	0.00	0.01	0.04	0.02
ODA as percent of GNI	−0.15*	−0.40*	0.21*	0.13	0.15	0.16*	0.14
Polity IV democracy index	− 0.10	− 0.41	0.36+	0.25+	0.39+	0.42*	0.42+
Polity IV durability	0.01	0.03	0.03	0.02	0.00	0.01	0.01
Civil war	−0.18	−0.88	2.02	0.36	1.23	1.93	0.41
International war	−1.56	−3.87	−3.80	− 1.08	0.76	0.88	0.56
Dependent variable	1.57***	1.23***	0.69***	0.66***	0.71***	0.70***	0.69***
Dependent variable in year t - 5	−0.60***	−0.29	− 0.03	0.08	0.12*	0.07	0.09
Country-years	607	607	515	554	554	553	554
R-squared	.98	.97	.61	.64	.72	.67	.71

*Note*. All regressions include year fixed effects and cluster-robust standard errors. 1990 ≤ t ≤ 1995

+ *p* < .10; * *p* < .05; ** *p* < .01; *** *p* < .001

Of course, these regressions may not fully address the potential selection effects discussed above. Including the current and lagged values of the dependent variable in the regressions controlled to some extent for a country’s prior commitment to improving child well-being. However, including those variables also reduced the statistical power, making it difficult to find significant relationships with CRC adoption. Missing data also reduced the statistical power; of the 940 possible country-years for these 164 countries from 1990 to 1995 (excluding 44 country-years when the countries were not in existence), the sample sizes in these regressions ranged from 515 to 607.

### Synthetic control analyses

Table 5 shows the results of our synthetic control analyses. As in the year fixed effect regressions, the relationships between CRC adoption and child mortality rates were never significant, but, at least for the first four years after adoption, they were in the expected direction; the positive coefficients in later years are not necessarily meaningful, because many of the control group countries had also adopted the CRC by then. The relationships between CRC adoption and vaccination rates were also almost always in the expected direction, as they were in the year fixed effect regressions. However, those relationships were often significant in the synthetic control analyses: CRC adoption had positive relationships with vaccination rates for all of these vaccines except DTP1, and those relationships remained significant for up to seven years after adoption. Focusing on the period from 1991 to 1993, the year in which some of the control group countries began to adopt the CRC, the effect sizes for mortality rates were small, with additional reductions in those rates averaging about 1 to 2 deaths per 1000 live births. The effect sizes for vaccination rates were larger, with additional increases in those rates averaging about 4 to 5%.

**Table 5:** Synthetic control estimates of average treatment effect of CRC adoption on child mortality and vaccination rates

Dependent variable	Average treatment effect estimated in:							
	1991	1992	1993	1994	1995	1996	1997	1998
Infant mortality rate	−1.87	−1.87	− 1.62	−1.22	−0.59	0.15	0.93	1.88
Under-5 mortality rate	−0.87	−1.07	− 0.87	−0.30	0.66	1.88	3.23	5.24
BCG vaccine rate	2.54*	4.99**	5.49*	2.78	3.72+	4.47	2.71	−0.97
DTP1 vaccine rate	1.36	3.04	3.53	1.92	1.23	1.98	2.60	1.84
DTP3 vaccine rate	5.66*	5.44+	6.71+	4.56	5.27	4.33	5.61	0.10
MCV1 vaccine rate	−0.13	0.97	11.19*	6.89	5.43	11.72*	7.73*	3.47
Polio3 vaccine rate	4.45+	4.82+	6.45+	2.96	3.95	5.55+	7.53*	3.24

*Note*. All analyses match countries that adopted the CRC in 1990 with synthetic control groups of countries that adopted the CRC after 1992, based on the average values of these predictor variables from 1985 to 1989: *ln (population)*, *ln (GDP* per capita*)*, *ODA as percent of GNI*, *Polity IV democracy index*, and the relevant dependent variable. The treatment groups include 43 and 41 countries for the mortality and vaccination analyses, respectively. The control groups are drawn from 21 and 18 countries for those analyses, respectively

+ *p* < .10; * *p* < .05; ** *p* < .01

The synthetic control analyses dropped countries that did not have each of the predictor variables in at least one of the years from 1985 to 1989. Doing so resulted in a significant decrease in the size of the treatment and control groups, especially the control group. Of the 56 countries in the initial treatment group, only 43 and 41 were included in the synthetic control analyses of mortality rates and vaccination rates, respectively. And of the 43 countries in the initial control group, only 21 and 18 were included in those respective analyses. Table 6 shows the countries in each group that were included and excluded from our synthetic control analyses.

**Table 6:** Countries included in and excluded from groups in synthetic control analyses

Analyses	Treatment group countries (adopted CRC in 1990)	Control group countries (adopted CRC after 1992)
Countries included in all synthetic control analyses	Angola, Argentina, Bangladesh, Benin, Bhutan, Bolivia, Brazil, Burkina Faso, Burundi, Chad, Chile, Costa Rica, Democratic Republic of the Congo, Ecuador, Egypt, El Salvador, Gambia, Ghana, Guatemala, Guinea, Honduras, Indonesia, Kenya, Mali, Mauritius, Mexico, Mongolia, Nepal, Nicaragua, Niger, Pakistan, Panama, Peru, Philippines, Sudan, Togo, Uganda, Uruguay, Venezuela, Viet Nam, Zimbabwe	Algeria, Botswana, Cameroon, Comoros, Congo, Fiji, Gabon, Iran, Iraq, Malaysia, Morocco, Mozambique, Oman, Papua New Guinea, Qatar, Saudi Arabia, Syrian Arab Republic, Turkey
Countries included in synthetic control analyses of mortality rates but not in synthetic control analyses of vaccination rates	Senegal, Sierra Leone	Liberia, Solomon Islands, Suriname
Countries excluded from all synthetic control analyses	Barbados, Belize, Democratic People’s Republic of Korea, Djibouti, Grenada, Guinea-Bissau, Namibia, Paraguay, Portugal, Romania, Russian Federation, Saint Kitts and Nevis, Seychelles	Afghanistan, Antigua and Barbuda, Cook Islands, Czech Republic, Greece, Haiti, Kiribati, Libya, Marshall Islands, Micronesia, Nauru, Saint Lucia, Saint Vincent and the Grenadines, Samoa, Slovakia, Somalia, South Africa, Swaziland, Tonga, Tuvalu, United Arab Emirates, Vanuatu

### Instrumental variable analyses

As a robustness check, we also conducted analyses using the two approaches that have most often been used in these studies to address the potential selection effects: instrumental variables and propensity score matching. We conducted several different instrumental variable analyses, using different combinations of instruments that have been used in prior studies. Table 7 presents the results of our analyses using the following five instruments: *Simmons ratification hurdles index*, *DPI federalism index*, *prior ICESCR adoption*, *prior CEDAW adoption*, and *prior regional CRC adoption percent*. The relationships of CRC adoption with mortality and vaccination rates never approached statistical significance in our instrumental variable analyses. Moreover, those relationships were in the opposite direction (with CRC adoption related to higher mortality rates and lower vaccination rates) more often than they were in the expected direction. Results with other combinations of instruments were generally similar to the results in Table 7.

**Table 7:** Instrumental variable regression estimates of relationship between CRC adoption and child mortality and vaccination rates

Variables included in regression in year t (except as specified)	Dependent variable in year t + 5						
	Infant mortality rate	Under-5 mortality rate	BCG vaccine rate	DTP1 vaccine rate	DTP3 vaccine rate	MCV1 vaccine rate	Polio3 vaccine rate
CRC adoption	−0.12	1.13	6.05	−1.88	−1.50	1.16	−2.11
Ln (population)	−1.12**	−2.54*	1.71**	1.45**	1.59*	1.84**	1.94**
Ln (GDP per capita)	−0.64	−1.77	3.82**	1.13	1.69	3.57*	2.18
Trade as percent of GDP	0.01	0.02	0.01	0.01	0.00	0.04	0.01
ODA as percent of GNI	−0.18*	−0.43*	0.15	0.05	0.10	0.08	0.10
Polity IV democracy index	−0.06	−0.36	0.02	0.20	0.32	0.23	0.38
Polity IV durability	0.01	0.03	0.01	0.01	−0.02	−0.01	− 0.01
Civil war	0.72	0.91	−0.51	−2.06	−1.64	−1.27	2.93
International war	−3.07+	−7.07+	−0.86	0.31	1.86	2.72	0.78
Dependent variable	1.50***	1.10***	0.65***	0.60***	0.76***	0.63***	0.69***
Dependent variable in year t-5	−0.54***	−0.18	− 0.07	0.05	0.05	0.04	0.05
Country-years	356	356	288	327	327	327	327
R-squared	.98	.97	.55	.55	.69	.61	.66

*Note*. All regressions use the following variables as instruments for CRC adoption: *Simmons ratification hurdles index*, *DPI federalism index*, *prior ICESCR adoption*, *prior CEDAW adoption*, and *prior regional CRC adoption percent*. All regressions include year fixed effects and cluster-robust standard errors. 1990 ≤ t ≤ 1995

+ *p* < .10; * *p* < .05; ** *p* < .01; *** *p* < .001

Unexpectedly, robust endogeneity tests comparing our year fixed effect regression and instrumental variable results consistently indicated that the instruments were unnecessary. This result suggests that our year fixed effect regressions may not be biased due to selection effects; the inclusion of the current period and lagged period values of the dependent variable may adequately control for the countries’ pre-existing commitments to improving child outcomes. Therefore, the main contribution of the synthetic control method may be the improvement in statistical power over the year fixed effect regressions, rather than the mitigation of selection effects.

### Propensity score analyses

For our propensity score analyses, we used the same predictor variables as for our synthetic control analyses and considered different combinations of numbers of matches per observation and calipers. Table 8 presents the results from our propensity score analyses that matched with a single nearest neighbour without any caliper. As in our instrumental variable analyses, the relationships of CRC adoption with mortality and vaccination rates never approached statistical significance in our propensity score analyses. And while those relationships were in the expected direction for four of the five vaccines in our propensity score analyses, they were almost always in the opposite direction for mortality rates and the BCG vaccination rate. Results with other combinations of numbers of matches per observation and calipers were generally similar to the results in Table 8.

**Table 8:** Propensity score estimates of average treatment effect of CRC adoption on child mortality and vaccination rates

Dependent variable	Average treatment effect estimated in:							
	1991	1992	1993	1994	1995	1996	1997	1998
Infant mortality rate	10.54	10.62	10.67	10.69	10.68	10.65	10.59	10.64
Under-5 mortality rate	27.04	27.10	27.10	26.92	26.70	26.36	25.87	25.73
BCG vaccine rate	−4.50	− 1.50	− 2.95	− 2.97	− 2.45	− 2.88	0.10	− 4.53
DTP1 vaccine rate	3.87	4.59	3.87	3.49	0.92	2.44	4.10	0.97
DTP3 vaccine rate	8.64	8.28	7.08	6.85	3.41	4.77	8.79	2.33
MCV1 vaccine rate	1.23	1.68	8.13	4.52	2.05	8.77	5.17	2.30
Polio3 vaccine rate	7.70	6.08	6.41	3.89	2.61	4.20	5.97	1.67

*Note*. All analyses match countries that adopted the CRC in 1990 with countries that adopted the CRC after 1992, using nearest neighbour, propensity score matching on these variables measured in 1990 – *ln (population)*, *ln (GDP* per capita*)*, *ODA as percent of GNI*, *Polity IV democracy index*, and the relevant dependent variable – and on the relevant dependent variable measured in 1985. The treatment groups include 43 and 42 countries for the mortality and vaccination analyses, respectively. The control groups include 20 and 19 countries for those analyses, respectively

Moreover, the advantages of using synthetic control methods for matching became apparent in our propensity score analyses, as the propensity score analyses tended to rely too heavily on a few control group countries for matching. For example, in the propensity score analysis of BCG vaccination rates that matched with a single nearest neighbour, the 42 treatment group countries were matched with only nine different control group countries and 25 of those 42 treatment group countries were matched with the same control group country (Turkey). But in the synthetic control analysis of BCG vaccination rates, 15 different countries were included in the synthetic control groups for the 41 treatment group countries and no control group country received a majority of the matching weight for more than 12 treatment group countries.

## Discussion

Scholars of international law and international relations have often questioned the effectiveness of international human rights treaties in changing countries’ behaviours [17–21, 33]. There are typically few penalties for noncompliance due to a lack of meaningful enforcement mechanisms and the unwillingness of the international community to use even those mechanisms that are available. Certainly, many countries adopt human rights treaties sincerely and make reasonable efforts to honour their commitments under the treaties. But other countries may ratify treaties strategically, with no intention of keeping their commitments, but merely to gain some benefit, such as admission to an international organization, or to avoid criticism for continuing human rights violations [26]. As summarized above, quantitative analyses of the effects of adoption of human rights treaties have often failed to support their effectiveness, although the results of those analyses have varied by treaty and analysis method.

To our knowledge, ours is only the second quantitative analysis of the effects of CRC adoption and the first analysis of the effects of adoption of any human rights treaty using synthetic control methods. Using instrumental variable methods, Simmons found weakly significant, beneficial effects of CRC ratification on MCV1 vaccination rates and no significant effects of CRC ratification on DTP3 vaccination rates [26]. Our year fixed effect analyses were generally consistent with those results, as we found positive relationships between CRC adoption and vaccination rates for those vaccines and the others that we considered, although those relationships never quite reached standard significance levels. However, in our synthetic control analyses, the relationships between CRC adoption and vaccination rates were often significant during the first seven years after adoption, despite the fact that our sample sizes were relatively small because of missing predictor variable data for many countries.

Even in our synthetic control analyses, we did not find any significant relationships between CRC adoption and child mortality rates. The distinction in our results between vaccination and mortality rates is not surprising. A country can improve its vaccination rates relatively quickly, by improving its efforts in areas such as professional knowledge exchanges, autonomy for its public health managers, and coordination with international agencies [48]. Child mortality is more complex, with a variety of proximate causes such as maternal age, parity, and birth interval; environmental contamination; nutrition; injury; and preventive health care, including vaccinations [49]. Therefore, one might not expect significant improvements even in infant mortality rates within the three- to five-year period after CRC adoption to which our analyses were effectively limited.

Djibouti’s experience illustrates the relative ease of increasing vaccination rates quickly, as compared with the challenge of making rapid improvements in mortality rates. In 1999, Djibouti’s vaccination rates for BCG, DTP3, MCV1, and Pol3 ranged from 23 to 26%, its infant mortality rate was 81 per 1000 live births, and its under-5 mortality rate was 103 per 1000 live births [2, 4]. In 2007, in its second periodic report submitted under the CRC, the Government of Djibouti described the measures it had taken to address those issues in the past decade, with assistance from UNICEF, the World Health Organization, the World Bank, and the United Nations Population Fund. Those measures included rehabilitating and refitting health-care facilities, establishing mobile surgery and primary health care clinics, making generic medicines available at affordable prices, training and placing gynaecologists and midwives in health-care centres, applying malnutrition protocols at health-care centres, adopting the Integrated Management of Childhood Illness programme, implementing a national immunization programme, launching a parental education programme, introducing a safe motherhood programme, and distributing mosquito nets to poor households [50]. Vaccination rates for each of those four vaccines (BCG, DTP3, MCV1, and Pol3) increased to at least 63% by 2003, representing an average increase of about 42% in just four years [4]. By comparison, infant and under-5 mortality rates decreased to 75 and 94, respectively, in 2003, representing decreases of only about 8% from 1999 [2]. In its 2012 annual report on Djibouti, UNICEF explained that efforts to reduce child mortality continued to face challenges such as high child malnutrition, high maternal mortality, very early pregnancies, and lack of access to improved sanitation facilities [51]. Djibouti has continued to make progress on reducing child mortality, with infant and under-5 mortality rates of 54 and 65, respectively, in 2015 [2], but UNICEF explains that child malnutrition, in particular, remains a major public health concern in Djibouti and contributes to about 35% of the causes of under-5 mortality [52].

The fact that both Simmons’ study and our study found some significant beneficial effects of the CRC is promising and suggests that the CRC has been important not merely as a normative statement of children’s rights, but also as a catalyst for real improvements in the lives of children in less developed countries. The 4 to 5% improvements in vaccination rates associated with CRC adoption that we found represented an additional five to seven million children vaccinated each year in less developed countries. Just for the vaccines considered in this study, those increases might have been associated with about 100,000 fewer deaths each year from diphtheria, tetanus, and pertussis, about 70,000 fewer deaths from measles, about 3000 fewer deaths from polio, and about 2000 fewer deaths from tuberculosis [53].

Our vaccination rate results are limited to the 59 less developed countries included in those analyses: countries that adopted the CRC either in 1990 or after 1992 and that had data available for the predictor variables prior to 1990. There is no reason to expect the time limitation to significantly affect the results; countries that adopted the CRC in 1991 or 1992 should not be very different than countries that adopted it earlier or later. The data limitation may be more significant, but the countries that were included and excluded from our vaccination rate analyses because of available predictor variable data were fairly well balanced by world region. The one exception to that balance is that only one of the seven applicable countries from Europe and Central Asia had sufficient predictor variable data available to be included in our vaccination rate analyses. Therefore, our results may not extend to countries in that region, but should generally apply to less developed countries from other regions.

Because some of the control group countries adopted the CRC as early as 1993, our results are also effectively limited to the period from 1991 until about 1995, when the difference between the two groups should be most meaningful. As noted above, this fact may have contributed to our inability to detect any significant relationships between CRC adoption and mortality rates, because any effects on mortality rates may take longer to materialize. Also, the fact that the treatment group countries had larger improvements in vaccination rates during the period from 1991 until 1997 does not necessarily mean that those differences would have persisted if the control group countries had never adopted the CRC.

A reviewer suggested that one limitation of our study is that we assumed that the data were normally distributed in the analysis for easier interpretation and that there may be a need to check the validity of this assumption; we do not believe that any such assumption was necessary for any of our analytical methods.

The limited variability in the timing of countries’ CRC adoptions and the lack of consistent, annual, country-level data prior to 1990 on most child indicators will make it challenging to further assess the effects of CRC adoption with quantitative methods. The CRC’s periodic reporting process should continue to enable qualitative analyses of the impact of CRC adoption, as all countries must report every five years on the actions they are taking to implement the CRC. The difficulty, of course, is that one can never be certain that the CRC is causing a country to take a particular action. However, beyond the CRC, our results suggest that it would be worthwhile to apply synthetic control methods to other human rights treaties, including studies of the effects of ICESCR and CEDAW adoption on health outcomes. Most other human rights treaties have much greater variability in countries’ adoption timing than the CRC does, which should allow even better synthetic control matching than in our analyses here. Perhaps other treaties will also fare better in synthetic control analyses than in instrumental variable or propensity score analyses, as the CRC did in this study.

Of course, researchers continue to develop new methods for these evaluations of binary policy interventions. Samartsidis et al. described the use of a latent factor model, or interactive effects model, which is a more flexible version of difference-in-difference modelling that allows the effect of unobserved confounder variables to vary over time [54]. They also explained Brodersen et al.’s causal impact model, which estimates a Bayesian model for the treated unit’s outcome that includes a time series component relating pre- and post-treatment outcomes for the treated unit and a regression component regressing post-treatment outcomes for the treated unit on pre-treatment outcomes for the control units to provide a counterfactual [55]. King, Lucas, and Nielsen introduced a matching frontier that allows researchers to choose solutions that provide the maximum possible predictor balance for any given sample size, without the need for the iterative process of parameter selection and balance checking that is typical of many matching methods [56]. And Doudchenko and Imbens proposed a new estimator that generalizes difference-in-difference, matching, and synthetic control methods [57]. Some of these approaches may also prove useful in estimating the effects of adoption of the CRC and other human rights treaties on health and other outcomes.

## Conclusions

In our preferred analyses using synthetic control methods, we find that CRC adoption may be related to additional reductions in infant and under-5 mortality rates of about 1 to 2 deaths per 1000 live births, on average, during the first three years after adoption, although those relationships are not statistically significant. And we find that CRC adoption is related to additional increases in vaccination rates for the five vaccines that we considered of about 4 to 5%, on average, during the first three years after adoption and that those relationships remain significant for up to seven years after adoption. These findings are important for both policy and research purposes. From a policy perspective, our results further support the effectiveness of adoption of core human rights treaties in promoting the right to health in less developed countries and, in particular, the effectiveness of CRC adoption in promoting children’s right to health. And from a research perspective, our results show the advantages of using synthetic control methods in studies of the effects of adoption of human rights treaties on health and other outcomes. Our analyses using other methods that have most commonly been used in these studies, including year fixed effect regressions, instrumental variables, and propensity score matching, did not find any consistent significant relationships between CRC adoption and mortality or vaccination rates.

## Data Availability

The dataset analysed during the current study is available in the Harvard Dataverse, https://dataverse.harvard.edu/dataset.xhtml?persistentId=doi:10.7910/DVN/C71CMD.

## Abbreviations

BCG: Baccille Calmette Guerin vaccine
CAT: Convention against Torture and Other Cruel, Inhuman or Degrading Treatment or Punishment
CEDAW: Convention on the Elimination of All Forms of Discrimination against Women
CERD: International Convention on the Elimination of All Forms of Racial Discrimination
CRC: Convention on the Rights of the Child
DTP3: Third dose of diphtheria, tetanus, and pertussis vaccine
ICCPR: International Covenant on Civil and Political Rights
ICESCR: International Convention on Economic, Social and Cultural Rights
MCV1: First dose of measles-containing-vaccine
Pol3: Third dose of polio vaccine
